# Mouse Spexin: (II) Functional Role as a Satiety Factor inhibiting Food Intake by Regulatory Actions Within the Hypothalamus

**DOI:** 10.3389/fendo.2021.681647

**Published:** 2021-07-02

**Authors:** Matthew K. H. Wong, Yuan Chen, Mulan He, Chengyuan Lin, Zhaoxiang Bian, Anderson O. L. Wong

**Affiliations:** ^1^ School of Biological Sciences, The University of Hong Kong, Hong Kong, Hong Kong; ^2^ School of Chinese Medicine, Hong Kong Baptist University, Hong Kong, Hong Kong

**Keywords:** spexin, GalR2/3, appetite control, glandular stomach, hypothalamus, mouse

## Abstract

Spexin (SPX) is a pleiotropic peptide with highly conserved protein sequence from fish to mammals and its biological actions are mediated by GalR2/GalR3 receptors expressed in target tissues. Recently, SPX has been confirmed to be a novel satiety factor in fish species but whether the peptide has a similar function in mammals is still unclear. Using the mouse as a model, the functional role of SPX in feeding control and the mechanisms involved were investigated. After food intake, serum SPX in mice could be up-regulated with elevations of transcript expression and tissue content of SPX in the glandular stomach but not in other tissues examined. As revealed by immunohistochemical staining, food intake also intensified SPX signals in the major cell types forming the gastric glands (including the foveolar cells, parietal cells, and chief cells) within the gastric mucosa of glandular stomach. Furthermore, IP injection of SPX was effective in reducing food intake with parallel attenuation in transcript expression of NPY, AgRP, NPY type 5 receptor (NPY5R), and ghrelin receptor (GHSR) in the hypothalamus, and these inhibitory effects could be blocked by GalR3 but not GalR2 antagonism. In agreement with the central actions of SPX, similar inhibition on feeding and hypothalamic expression of NPY, AgRP, NPY5R, and GHSR could also be noted with ICV injection of SPX. In the same study, in contrast to the drop in NPY5R and GHSR, SPX treatment could induce parallel rises of transcript expression of leptin receptor (LepR) and melanocortin 4 receptor (MC4R) in the hypothalamus. These findings, as a whole, suggest that the role of SPX as a satiety factor is well conserved in the mouse. Apparently, food intake can induce SPX production in glandular stomach and contribute to the postprandial rise of SPX in circulation. Through GalR3 activation, this SPX signal can act within the hypothalamus to trigger feedback inhibition on feeding by differential modulation of feeding regulators (NPY and AgRP) and their receptors (NPY5R, GHSR, LepR, and MC4R) involved in the feeding circuitry within the CNS.

## Introduction

Spexin (SPX) is a neuropeptide first identified using the hidden-Markov model for data mining of novel peptides in human proteome (1, 2). Its mature peptide is highly conserved with only 1 to 2 a.a. substitutions from fish to mammals (3, 4). It is widely expressed in different tissues, including the liver, pancreas, visceral fat, intestine, and adrenal gland, e.g., in the rat (5) and human (6), and reported to have regulatory actions on gut motility (7), stomach contraction (1), bile acid synthesis (8), food consumption (9, 10), glucose and lipid homeostasis (11, 12), fatty acid uptake (13, 14), hormone secretion (15, 16), locomotor activity (14), nociception (17, 18), stress/anxiety responses (19), and cardiovascular/renal functions (20). In human, SPX is encoded by the gene c12orf39 located in chromosome 12 (21) and abnormalities in SPX expression/serum levels can be associated with childhood (22, 23) and adult obesity (24), type I/II diabetes (6, 25), gestational diabetes (26), metabolic syndrome (27), cardiovascular disease (28), and psychiatric disorders including anorexia nervosa (29) and anxiety/depression (30, 31). Recently, SPX has emerged as a new target for drug design/therapeutic development (32), e.g., for SPX-based analogs with biased agonism for GalR2 signaling (33, 34) and anxiolytic/antidepressive activity (35).

Regarding the functional role of SPX in feeding control, mainly based our studies in goldfish, SPX was confirmed to be a novel satiety factor in fish models [for review, see (4)]. In goldfish, food intake could up-regulate plasma SPX level with parallel rises of SPX expression in the liver (36) and brain areas, including the telencephalon, hypothalamus, and optic tectum (9), which are key structures within the CNS for feeding control in fish species (37). The SPX responses caused by feeding were mimicked by IP injection of glucose and insulin, respectively, and the stimulatory effects of glucose could be blocked by insulin antagonists or inactivation of insulin receptor (36). Furthermore, SPX treatment by IP/ICV injection was effective in reducing food intake by inhibiting feeding behavior in goldfish, which could be correlated with the drops in NPY and AgRP with parallel rises of POMC, CCK, and CART signals in brain areas involved in feeding control (9). Our results imply that the postprandial responses of SPX were caused by insulin release after glucose uptake and contributed to the satiation response after feeding, which was probably mediated by SPX regulation of orexigenic/anorexigenic signals expressed in different brain areas with the feeding circuitry. Our findings on the association of central expression of SPX with food intake, as well as the effects of SPX in regulating the feeding signals expressed in the brain (e.g., AgRP and POMC) have been recently confirmed in other fish species, e.g., in zebrafish (10), grouper (38), ya fish (39), tongue sole (40), and spotted scat (41). Although SPX is known to be co-evolved with galanin and its biological actions have been proposed to be mediated by the galanin receptors GalR2 and GalR3 (42), the receptor(s) mediating the feeding responses of SPX has not been defined and it is still unclear if the role of SPX as a satiety factor can also be observed in higher vertebrates, including the mammals.

In this study, using the mouse as a representative model for mammals, the functional role of SPX in feeding control and the underlying mechanisms involved were investigated. To focus on the role of SPX as a satiety factor, experiments were conducted to (i) examine the effects of food intake in mice on SPX release into circulation and SPX expression (both at transcript and protein levels) in tissues with a potential role in metabolic control/feeding regulation; (ii) study the *in vivo* action of SPX (via peripheral administration by IP injection/central administration by ICV injection) on food consumption with possible involvement of GalR2 and GalR3; and (iii) elucidate the “central mechanisms” of SPX in regulating food intake (by ICV injection) by altering the expression of feeding regulators and/or their receptors at the level of the hypothalamus, which is known to be a major area in the brain with the neuronal circuitry for appetite control (43). Our results reveal that the role of SPX as a satiety factor is well conserved in the mouse, and SPX regulation of feeding is mediated by GalR3 activation coupled to differential modulation of feeding regulators and their receptors expressed in the hypothalamus. Interestingly, the glandular stomach may represent a major source of the postprandial signal of SPX in mouse model, which is quite different from the findings reported in fish species.

## Materials and Methods

### Animals, Housing Entrainment, and Tissue Sampling

Mice (*Mus musculus*) of the C57BL/6N strain (male only) with body weight of 20-25 g were housed by individual caging at 22°C and 60% relative humidity under a daily cycle of reversed photoperiod (12 h dark:12 h light with light off and food provision at 10:00 am) and fed *ad libitum* with standard chow for rodents (PicoLab^®^ Rodent Diet 20/5053; Labdiet, St. Louis, USA). Female animals were not used to avoid fluctuation of estrogen during the estrous cycle, as estrogen is known to be anorexigenic during the pre-ovulation phase in rodents (44). The mice were routinely switched from group caging (four animals per cage) under normal photoperiod to individual caging under reversed photoperiod 20 days after their arrival and acclimated for ≥ 10 days prior to feeding experiments or SPX treatment by IP/ICV injection. Based on our validation, body weight and food intake were stabilized in mice 5 days after switching to the new conditions under individual housing (**Supplemental Figures 1A, B**). In our studies, the animals were anesthetized by co-treatment of xylazine (10 mg/kg) and ketamine (10 mg/kg) after food intake/SPX injection. Blood samples were harvested by saphenous puncture in the hind limb followed by decapitation of the animal for tissue collection for real-time PCR of target genes/SPX measurement by ELISA assay according to the protocol approved by the Committee on the Use of Live Animal in Teaching and Research at the University of Hong Kong (Hong Kong).

### Food Intake on SPX Release and Tissue Expression

After the mice had been entrained to individual caging under reversed photoperiod, they were divided into the “fed” group (with food provision at 10:00 am) and “unfed” group (without food provision) with 12 animals per group for different time points as indicated. Blood samples and fresh tissues, including the liver, pancreas, omental fat, forestomach, glandular stomach, different parts of the gut, hypothalamus, and pituitary, were harvested at different time points (with the time for food provision as “zero hour”). The blood samples were used for serum preparation for measuring SPX released into circulation using a mouse SPX ELISA kit (EK-02-81, Phoenix Pharmaceuticals) while the tissues collected were subjected to total RNA extraction with TRIZOL (Sigma-Aldrich), reverse transcription by Superscript II (Invitrogen) in the presence of DNase I (to remove possible genomic DNA contamination), and real-time PCR for SPX transcript expression using a QuantiTect SYBR Green RT-PCR Kit (Qiagen, Hilden, Germany). Real-time PCR was conducted in a RotorGene qPCR system (Qiagen) with primers for SPX and PCR conditions as described in **Table 1**. The authenticity of PCR product (109 bp in size with *Tm* at 84°C) was routinely confirmed by melting curve analysis after the assay. Serial dilutions of plasmid DNA with SPX sequence were used as the standards for data calibration. GAPDH, an internal control proven to be appropriate for tissue expression profiling using RT-PCR, was shown to be altered in the liver and white fat as a result of feeding. As a result, β actin, which was confirmed to be not affected by feeding and SPX treatment, was used as the internal control for data normalization. To evaluate the effects of food intake on SPX expression at protein level, tissue lysate was prepared from selected tissues with ice-cold PBS using a LT TissueLyser (Qiagen) and subjected to SPX measurement using SPX ELISA. To adjust for variations in the amount of tissue used in lysate preparation, parallel measurement of protein content in these samples using a BCA Protein Assay Kit (ThermoFisher Scientific) were used as the normalization control. To shed light on the effects of food intake on histological expression of SPX at the tissue level, omental fat, forestomach, and glandular stomach harvested by the end of the feeding experiment were fixed in Zamboni fixative followed by the standard procedures for ethanol dehydration and paraffin embedding. Tissue sections (10 µm in thickness) were then prepared and used for immunohistochemical staining (IHS) with a VectaStain Elite ABC Kit (Vector Lab) using a SPX antiserum (EK-02-81, Phoenix Pharmaceuticals; 1:600, 15 h at 4°C) previously validated to be specific for mouse SPX (see Part I of our 3-paper series). For semi-quantitative analysis of the signals for SPX immunostaining, the duration of incubation with second antibody (goat anti-rabbit, 1:400) and signal development using diaminobenzidine (Sigma) as the substrate was routinely fixed at 2 h and 30 min, respectively, with parallel staining of a similar dilution of normal rabbit serum (NRS) as the negative control.

**Table 1:** Primer sequences and PCR conditions for real-time PCR assays in mouse.

Gene Target/GenBank accession no.	Real-time PCR Condition					Product size, *Tm* value &
Sequences of forward (F) & reverse primers (R)	Denaturing	Annealing	Extension	Detection	Cycle	PCR efficiency
AGRP/NM_007427.2						
F: 5′-ATGCTGACTGCAATGTTGCTG-3′	94°C	57°C	72°C	80°C	× 34	141 bp, 87°C
R: 5′-CAGACTTAGACCTGGGAACTCT-3′	30 s	30 s	30 s	20 s		& 99.8%
β- actin/NM_007393.3						
F: 5′-CATCTTGGCCTCACTGTCCAC-3′	94°C	57°C	72°C	75°C	× 35	69 bp, 81°C
R: 5′-GGGCCGGACTCATCGTACT-3′	30 s	30 s	30 s	20 s		& 100.0%
CART/NM_001081493.1						
F: 5′-GATCGGGAAGCTGTGTGACT-3′	94°C	57°C	72°C	78°C	× 35	177 bp, 82°C
R: 5′-TGAGGGGAACGCAAACTTTA-3′	30 s	30 s	30 s	20 s		& 99.2%
CRH/NM_205769.2						
F: 5′-AAACAGCGTTATTTGTATTGCC-3′	94°C	57°C	72°C	75°C	× 35	189 bp, 82°C
R: 5′-CATTTCGTCCTAGCCACCC-3′	30 s	30 s	30 s	20 s		& 100.0%
Ghrelin/NM_021488.1						
F: 5′-CCATCTGCAGTTTGCTGCTA-3′	94°C	57°C	72°C	80°C	× 40	273 bp, 87°C
R: 5′-GCTTGTCCTCTGTCCTCTGG-3′	30 s	30 s	30 s	20 s		& 98.3%
GHSR/NM_177330.4						
F: 5′-TGGAGATCGCGCAGATCAG-3′	94°C	57°C	72°C	76°C	× 40	188 bp, 82°C
R: 5′-CCGGGAACTCTCATCCTTCAG-3′	30 s	30 s	30 s	20 s		& 100.0%
LEPR/NM_001122899.1						
F: 5′-AGTCTTCGGGGATGTGAATG-3′	94°C	57°C	72°C	78°C	× 45	214 bp, 85°C
R: 5′-TTTGGCTGTCCCAAGAAATC-3′	30 s	30 s	30 s	20 s		& 100.0%
Leptin/NM_008493.3						
F: 5′-GAGACCCCTGTGTCGGTTC-3′	94°C	57°C	72°C	76°C	× 35	139 bp, 81°C
R: 5′-CTGCGTGTGTGAAATGTCATTG-3′	30 s	30 s	30 s	20 s		& 100.0%
MC4R/NM_016977.3						
F: 5′-CCCGGACGGAGGATGCTAT-3′	94°C	57°C	72°C	75°C	× 40	101 bp, 80°C
R: 5′-TCGCCACGATCACTAGAATGT-3′	30 s	30 s	30 s	20 s		& 99.8%
MCH/NM_029249.2						
F: 5′-GTCTGGCTGTAAAACCTTACCTC-3′	94°C	57°C	72°C	75°C	× 35	161 bp, 81°C
R: 5′-CCTGAGCATGTCAAAATCTCTCC-3′	30 s	30 s	30 s	20 s		& 100.0%
NPY/NM_023456.3						
F: 5′-TACTCCGCTCTGCGACACTA-3′	94°C	57°C	72°C	75°C	× 35	150 bp, 81°C
R: 5′-TCACCACATGGAAGGGTCTT-3′	30 s	30 s	30 s	20 s		& 100.0%
NPY5R/NM_016708.3						
F: 5′-TTTGTCACGGAGAACAATACTGC-3′	94°C	57°C	72°C	72°C	× 40	238 bp, 86°C
R: 5′-TGCGCTTTTTCATAACAGCCAT-3′	30 s	30 s	30 s	20 s		& 99.0%
POMC/NM_008895.3						
F: 5′-ATGCCGAGATTCTGCTACAGT-3′	94°C	57°C	72°C	80°C	× 40	170 bp, 86°C
R: 5′-TCCAGCGAGAGGTCGAGTTT-3′	30 s	30 s	30 s	20 s		& 100.0%
SPX/AK043509.1						
F: 5′-AATAAGGAGGGAGGCAAGGA-3′	94°C	57°C	72°C	78°C	× 40	109 bp, 80°C
R: 5′-GACCTTCCAGCAGTTTCAGC-3′	30 s	30 s	30 s	20 s		& 98.9%

(Primer sequences were designed using Primer-Blast in NCBI with the setting for annealing temperature of 55–65°C, product size ≤ 300 bp, and selecting primers spinning an exon-exon junction).

### IP injection of SPX on Food Intake and Gene Expression in the Hypothalamus

To investigate the effect of SPX on food intake, mouse SPX (NWTPQAMLYLKGAQ, 98.2% pure) was synthesized by GenScript (Piscataway, NJ) and used in the mice entrained to the new housing conditions for both time course and dose dependence studies with peripheral administration of SPX by IP injection. Based on our validation, the mice under “single caging” with a reversed photoperiod could still retain the diurnal rhythm of feeding, with cumulative rises of food intake during the dark phase and little/no feeding during the light phase (**Supplemental Figure 1C**). The rate of food consumption averaged over the duration of feeding (in “g/h”) was found to the highest during the first hour in the dark phase, reduced to a lower level up to 12 h, and dropped gradually in the light phase until 24 h (**Supplemental Figure 1D**). By fixing the feeding period to the first hour in the dark phase, repeated saline injections (5 µl/g BW, IP with 5-min intervals starting at 10:00 am) up to two times did not alter food intake compared to the control (without injection) but food consumption was reduced by three consecutive IP injections, (**Supplementary Figure 1E**). Furthermore, the 24-h food consumption was not affected by saline injection (one injection/day) up to 4 days although a drop in food intake was noted with daily IP injection up to 6 days (**Supplementary Figure 1F**). Based on these findings, IP injection of SPX (5 µl/g BW) was conducted at 10:00 am (i.e., the time for light off) with one injection per day and food consumption within the first hour during the dark phase was used as an index for feeding responses. To test if the effect on feeding can lead to a similar change in body weight, the weight gain over a 3-day period with daily injection of SPX was also examined. In our studies, saline injection was used as the control while parallel treatment with mouse leptin (Sigma) was conducted to serve as a “positive control” for the feeding responses and body growth.

### Receptor Specificity for the Feeding Responses Induced by SPX

To shed light on the receptor specificity of GalR2/3 in the feeding responses induced by SPX, tissue expression of GalR2 and GalR3, especially in the brain and hypothalamus, were monitored by RT-PCR (for transcription expression) and Western blot (for protein expression). RT-PCR was conducted using primers for mouse GalR2 and GalR3 with PCR conditions as described in **Table 2** and parallel PCR for GAPDH was used as the internal control. For Western blot, tissue lysate prepared was subjected to SDS-PAGE and transblotted onto a PVDF membrane followed by immunoblotting using the antibodies for mouse GalR2 and GalR3, respectively (Alomone Labs, 1:8000). The specificity of signals detected was confirmed by antibody preabsorption with the synthetic peptides for GalR2/3 (10 mM, 15 h at 4°C) used as the antigens for the two targets provided by the company. Parallel blotting for β actin was also conducted using an Actin AB-1 Kit (Calbiochem) to serve as a loading control. To unveil the receptor subtype involved in the feeding responses by SPX, the effect on food intake induced by IP injection of SPX was examined with co-treatment of the GalR2 antagonist M871 or GalR3 antagonist SNAP 378890 (SNAP) (Tocris Bioscience). In parallel experiment, IP injection of dN1-Qu (synthesized by Genscript, 97.8% pure), a galanin mutant specific for GalR2 activation (33), was also performed to test if the treatment can mimic the effects of SPX. In these studies, after obtaining the data for food intake during the first hour in the dark phase, the hypothalamus was excised for real-time PCR targeting various feeding regulators and their receptors known to play a role in central regulation of food intake in mouse with primers and PCR conditions as described in **Table 1**. Serial dilutions of plasmid DNA with the amplicons for respective gene targets were used as the standards, and parallel measurement of transcript expression of β actin was used as the internal control.

**Table 2:** Primer sequences and PCR conditions for RT-PCR of mouse GALR2/3.

Gene target/GenBank accession no	PCR condition				Product size
Sequences of forward (F) and reverse primers (R)	Denaturing	Annealing	Extension	Cycle no.
GALR2/NM_010254.4					
F: 5′ CCCTCGATCCAGCCTGTTAAA 3′	94°C	64°C	72°C	×45	113bp
R: 5′ CGGTCTTTAGCTGCAACCCT 3′	45 s	30 s	60 s		
GALR3/AF042783					
F: 5′ ACCAAGGAGCATCTAGGGCA 3′	94°C	64°C	72°C	×40	111bp
R: 5′ CCCTGTGTTTAGTTGGGTGGA 3′	45 s	30 s	60 s		
GAPDH/NM_008084.3					
F: 5′ AACTTTGGCATTGTGGAAGG 3′	94°C	64°C	72°C	×40	223bp
R: 5′ ACACATTGGGGGTAGGAACA 3′`	45 s	30 s	60 s		

### Central Actions of SPX on Food Consumption and Gene Expression in the Hypothalamus

To confirm that the feeding responses caused by SPX indeed were mediated by central actions within the brain, ICV injection of SPX was conducted in the mouse for time course/dose dependence studies as described previously (45). Briefly, a 21-gauge cannula was installed on the skull of the mice acclimated to individual caging and reversed photoperiod with the tip positioned in the lateral ventricle using a SR-5M stereotaxic instrument (Narishige Group; with 0.5 mm posterior to the bregma, 1.0 mm lateral to the midline, and 2.0 mm below the skull surface). The cannula was secured on the skull by dental cement and sealed with a stylet to prevent leaking of cerebrospinal fluid (CSF) and potential infection in the CNS. The animals were allowed to recover for 5 days prior to ICV injection. On the day of experiment, the stylet was removed and appropriate levels of SPX prepared in sterilized artificial CSF (aCSF, Alzet, Cupertino, CA) were injected into the lateral ventricle (5 µl/animal given at 10:00 am) by a 10-µl Hamilton syringe connected to an injector with a blunt-end needle fitted within the cannula. After that, the animal was returned to the cage for feeding study during the dark phase. In parallel experiment, the mice were sacrificed at different time points after SPX treatment for harvesting the hypothalamus for real-time PCR of the respective gene targets involved in feeding control as described in the preceding section. In these studies, parallel ICV injection of aCSF was used as the control treatment.

### Data Normalization and Statistical Analysis

For transcript expression measured by real-time PCR, standard curves constructed by serial dilutions of plasmid DNA carrying the ORF/amplicons of the respective gene targets with a dynamic range of ≥ 10^5^, amplification efficiency ≥ 0.98, and correlation coefficient of ≥ 0.95 were used for data calibration with the RotorGene Q-Rex software (Qiagen). To control for different amount of tissues used in RNA extraction, the raw data for qPCR (in femtomole target transcript detected) were normalized as a ratio of β actin mRNA expressed in the same sample. The data obtained were then transformed as a percentage of the mean value in the control group for statistical analysis (as “% Ctrl”). The data presented are expressed as mean ± SEM (N = 12) and subjected to one-way ANOVA followed by Newman-Keuls test (for food consumption and gene expression with agonist/antagonist treatment) or two-way ANOVA followed by Bonferroni test (for time course studies with food intake/SPX treatment). Statistical analyses were performed using Prism version 5.0 (GraphPad, San Diego, CA), and the difference between groups was considered as significant when *p* < 0.05.

## Results

### Effects of Food Intake on SPX Release and Tissue Expression

**Figure 1:**
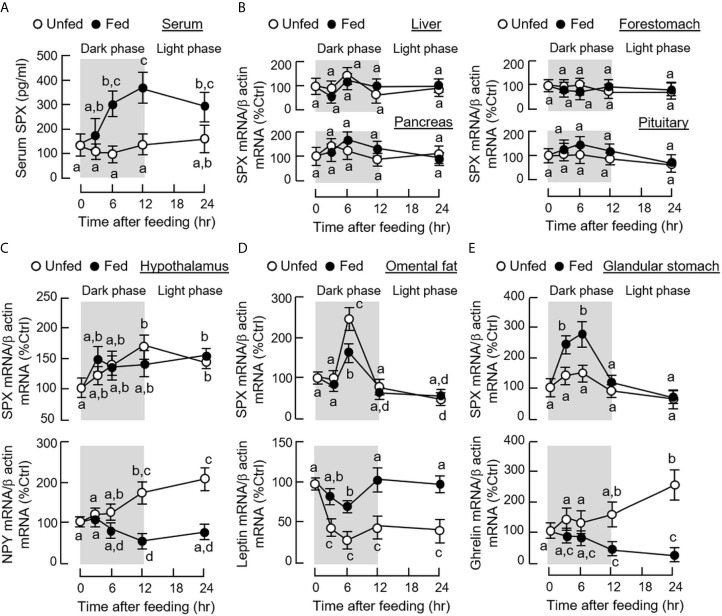
Effects of food intake on SPX released into circulation and SPX gene expression at tissue level in mice. After acclimation to inverted photoperiod, one group of the mice was maintained under the scheduled feeding plan (as the “fed” group) while the other group did not receive the supply of chow pellets (as the “unfed” group). Serum samples and target tissues were harvested at the time points as indicated (with light off at 10:00 am as time “zero”). Time course of food intake on SPX released into circulation was examined by measuring SPX levels in serum samples using SPX ELISA **(A)**. The corresponding effects of food intake on SPX mRNA expression in target tissues, including the liver, pancreas, forestomach and pituitary, was quantitated by real-time PCR **(B)**. In selected tissues, including the hypothalamus **(C)**, omental fat **(D)** and glandular stomach **(E)**, besides SPX mRNA, parallel expression of NPY, leptin and ghrelin transcript levels in the respective tissues were also monitored to serve as positive control. For the data presented, the groups denoted by different letters represent a significant difference at *p* < 0.05.

To establish the functional link of SPX with feeding in the mouse, the effects of food intake on SPX release into circulation and SPX expression at tissue level were examined. As shown in **Figure 1A**, food intake in the mice during the dark phase could elevate serum levels of SPX in a time-dependent manner with peak responses occurred during 6 to 12 h after the initiation of feeding. Parallel measurement of SPX mRNA in the liver, pancreas, forestomach, and pituitary did not reveal significant changes during the same period (**Figure 1B**). The same was also true for SPX gene expression in the adrenal gland, spleen, and different regions of the intestine (data not shown). In the hypothalamus, a gradual rise in NPY mRNA was noted in the unfed group while food intake was effective in suppressing NPY gene expression (the positive control for feeding responses in CNS). Similar to other tissues examined, food consumption did not alter transcript expression of SPX at the hypothalamic level (**Figure 1C**). Of note, food intake during the dark phase was found to have differential effects on SPX expression in the omental fat (**Figure 1D**) and glandular stomach (**Figure 1E**). For the omental fat, a transient rise in SPX mRNA level was noted at 6 h in the unfed group, which occurred during the rapid drop in leptin mRNA without food provision in the dark phase. This SPX response was down-regulated by food intake with a parallel elevation in leptin transcript expression in the fed group (the positive control for feeding response in white adipose tissue) (**Figure 1D**). In glandular stomach, unlike the inhibitory action in omental fat, food intake during the dark phase was effective in triggering a transient rise in SPX transcript expression with the peak responses occurred 3 to 6 h after the initiation of feeding. Meanwhile, a gradual drop in ghrelin mRNA level was also observed up to 24 h after food consumption (the positive control for feeding response in the stomach) (**Figure 1E**).

**Figure 2:**
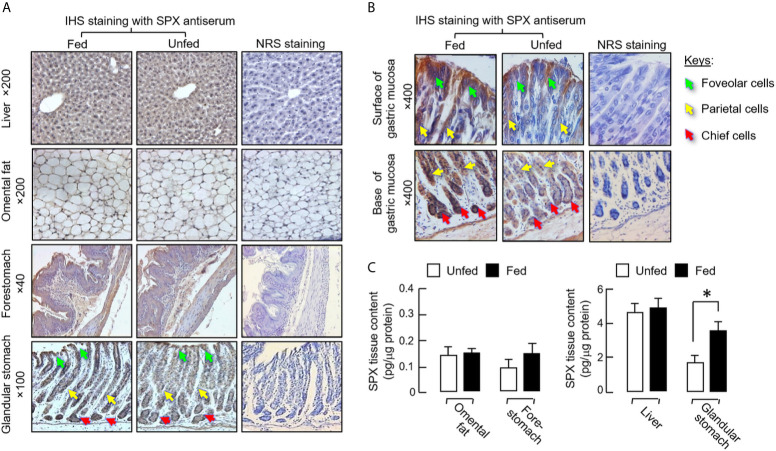
Effect of food intake on protein expression of SPX at tissue level in mice. The feeding experiment was conducted as described in the preceding section with one group as the “fed” group (with food provision at 10:00 am) and the other as the “unfed” group (deprived of food supply). After 6 h with the respective feeding protocols, the liver, omental fat, forestomach and glandular stomach were excised and subjected to IHS staining with a SPX anti-serum (1:600) raised in rabbit **(A)**. Counter staining with hematoxylin was routinely performed and parallel staining with normal rabbit serum (NRS, 1:600) was used as the negative control. Notable changes in SPX staining were not apparent in the liver, omental fat and forestomach but intensification of SPX signals was noted in gastric mucosa of glandular stomach with food intake, especially in the foveolar cells, parietal cells and chief cells of the gastric glands **(B)**. In the same study, tissue lysate was also prepared from the samples freshly collected in the respective tissues and used for measurement of tissue SPX content **(C)**. In the graph presented, the group denoted by an asterisk (*) represents a significant different at *p* < 0.05 compared to the respective control.

Given that SPX immunostaining were identified in mouse tissues involved in metabolic control and endocrine functions (see Part I of this three-paper series), IHS staining with SPX antiserum was conducted in tissue sections prepared from the liver, omental fat, forestomach, and glandular stomach of the mice after 6 h in the dark phase with/without food provision (**Figure 2A**). In this case, food intake did not induce major changes in SPX immunostaining in the liver, omental fat, and forestomach, although the intensity of SPX signals appeared to be elevated in the glandular stomach, especially on the surface of gastric mucosa with foveolar cells and in the lower region of the mucosal layer with parietal cells and chief cells forming the gastric glands (**Figure 2B**). Apparently, noticeable changes in SPX immunostaining were not observed in the muscle layers of forestomach and glandular stomach. Given that IHS staining is not ideal for quantitation of protein signals, tissue contents of SPX were also monitored using a SPX ELISA in the same study. In this case, consistent with the results of IHS staining, protein expression of SPX was found to be up-regulated in glandular stomach after feeding and similar changes were not observed in the liver, omental fat and forestomach (**Figure 2C**).

### Effects of IP Injection of SPX on Food Intake and Gene Expression in the Hypothalamus

**Figure 3:**
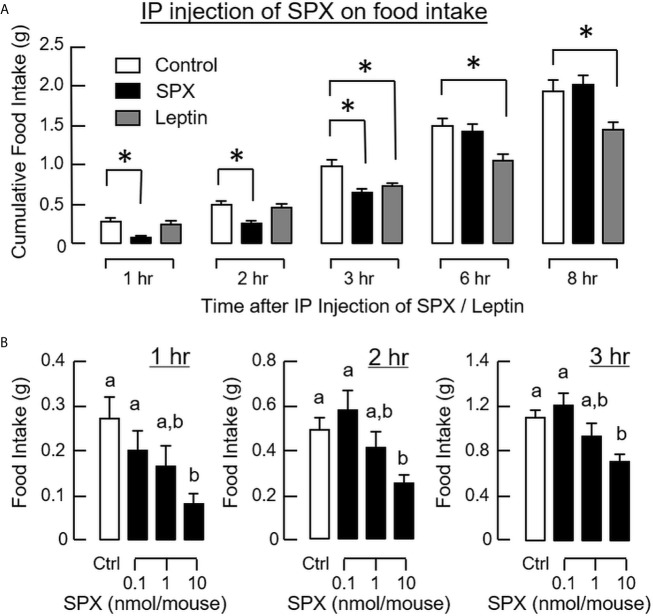
Peripheral administration of SPX on food intake in mice. Time course **(A)** and dose dependence **(B)** of SPX treatment by IP injection (5 µl/g BW, injected at 10:00 am) on food consumption in mice. For time course study, SPX was tested at a dose of 10 nmol/mouse to examine its effects on food intake up to 8 h. For dose dependence, the corresponding effects of increasing levels of SPX (0.1-10 nmol/mouse) were monitored for 1, 2, and 3 h, respectively. IP injection with physiological saline was used as the control treatment and parallel injection with leptin (10 nmol/mouse) was conducted to serve as a positive control. The groups denoted by asterisks (*)/different letters represent a significant difference at *p* < 0.05.

To investigate the effects of SPX on food intake, IP injection of SPX (10 nmol/mouse) was performed in the mice at the beginning of the dark phase (at 10:00 am) with leptin treatment as the parallel control. As shown in **Figure 3A**, a transient reduction in food consumption was observed up to 3 h after the initiation of feeding. In contrast, the drop in food intake caused by IP injection of leptin (10 nmol/mouse, the positive control for feeding response) could be noted after 3 h and maintained up to 8 h in our feeding experiment. In the first 3 h during the dark phase, increasing levels of SPX was effective in reducing food intake in a dose-dependent manner (**Figure 3B**). Of note, unlike the inhibitory effect of leptin, SPX was not able to reduce the cumulative food intake over a 24-h period (**Supplemental Figure 2A**), suggesting that “compensatory feeding” might have occurred during the latter half of dark phase/in the light phase. Interestingly enough, daily injection of SPX and leptin up to 3 days were both effective in reducing the weight gain occurred during the same period (**Supplemental Figure 2B**).

**Figure 4:**
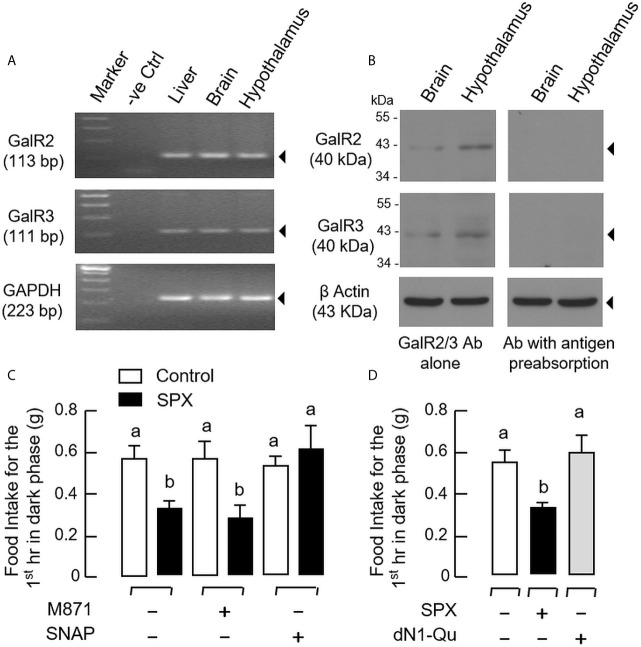
Receptor specificity for SPX inhibition on food consumption in mice. **(A)** RT-PCR for GalR2 and GalR3 expression in the liver, brain and hypothalamus. Total RNA isolated from target tissues was used for RT-PCR with primers for GalR2 and GalR3, respectively, with GAPDH as internal control and PCR with no template as negative control (“-ve Ctrl”). **(B)** Western blot of GalR2 and GalR3 expression in the brain and hypothalamus. Tissue lysate was prepared from mouse brain and hypothalamus and subjected to SDS- PAGE and Western blot using the antibody (Ab) for GalR2 and GalR3, respectively, with β actin as loading control. To confirm the specificity of immunoblotting, Western blot was also conducted with antibodies pre-absorbed with the synthetic peptides for GalR2/3 used to raise the antibody provided by the company. **(C)** GalR2 and GalR3 blockade on the inhibitory effect of SPX on food intake in mice. IP injection of SPX (5 nmol/mouse) was performed at 10:00 am with/without co-treatment of the GalR2 antagonist M871 (50 nmol/mouse) or GalR3 antagonist SNAP (50 nmol/mouse) to test the effect on food consumption occurred during the first hour in dark phase. **(D)** Effect of GalR2 activation on food consumption in mice. In parallel experiment, IP injection of the GalR2 agonist dN1-Qu (10 nmol/mouse) was conducted with SPX treatment (10 nmol/mouse) as positive control. For the data presented, the groups denoted by different letters represent a significant difference at *p* < 0.05.

**Figure 5:**
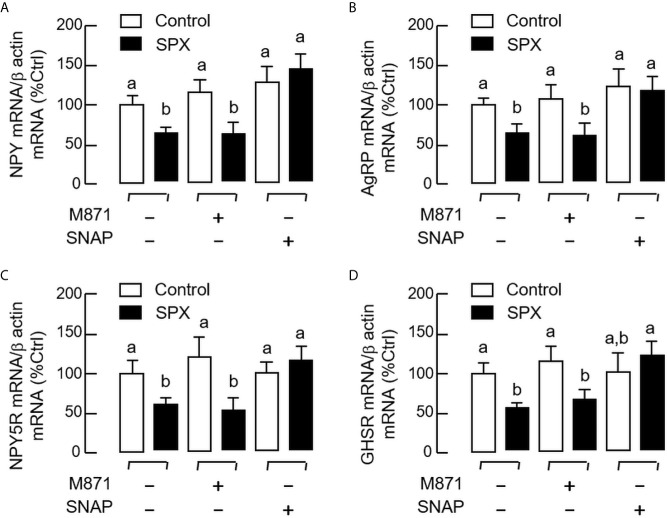
Receptor specificity for SPX regulation of hypothalamic expression of orexigenic factors and their receptors. IP injection of SPX (5 nmol/mouse) was performed in mice with/without the co-treatment of the GalR2 antagonist M871 (50 nmol/mouse) or GalR3 antagonist SNAP (50 nmol/mouse). The hypothalamus was harvested one hour later after IP injection. Total RNA was isolated and subjected to real-time PCR for transcript expression of NPY **(A)**, AgRP **(B)**, NPY5R **(C)** and GHSR **(D)**, respectively with β actin mRNA as internal control. The groups denoted by different letters represent a significant difference at *p* < 0.05.

To shed light on the receptor specificity for the feeding response of SPX, RT-PCR for GalR2 and GalR3 was also performed in selected tissues, including the liver, brain, and hypothalamus. In this case, transcript signals for the two receptors were consistently detected in all the tissues examined (**Figure 4A**). These results are consistent with the parallel studies using Western blot, in which the protein signals of GalR2 and GalR3 could be identified in tissue lysates prepared from the brain and hypothalamus using the antibodies targeting the respective receptors (**Figure 4B**). To define the receptor subtype responsible for feeding inhibition by SPX, IP injection of SPX was conducted with co-treatment of the GalR2 antagonist M871 and GalR3 antagonist SNAP, respectively. As shown in **Figure 4C**, co-treatment with SNAP could revert the inhibitory effect of SPX on food consumption but M871 was not effective in this regard. In parallel experiment, IP injection of the GalR2 agonist dN1-Qu, a quadruple mutant of galanin with a.a. substitutions based on SPX sequence (33), was not able to mimic the feeding inhibition by SPX (**Figure 4D**) (GalR3 agonist was not examined in our study as the agonists with GalR3 selectivity are not yet available). In the study with antagonist co-treatment, the hypothalamus was also harvested after the feeding study to investigate the possible effects on central expression of feeding regulators/their receptors. Besides the feeding responses observed, SPX treatment was found to down-regulate the transcript expression of NPY (**Figure 5A**), AgRP (**Figure 5B**), NPY type 5 receptor (NPY5R, **Figure 5C**), and ghrelin receptor (GHSR, **Figure 5D**) at the hypothalamic level. Similar to the results of food intake, these inhibitory effects could be blocked by co-treatment with the GalR3 antagonist SNAP but not the GalR2 antagonist M871.

### Effects of ICV Injection of SPX on Food Intake and Gene Expression in the Hypothalamus

**Figure 6:**
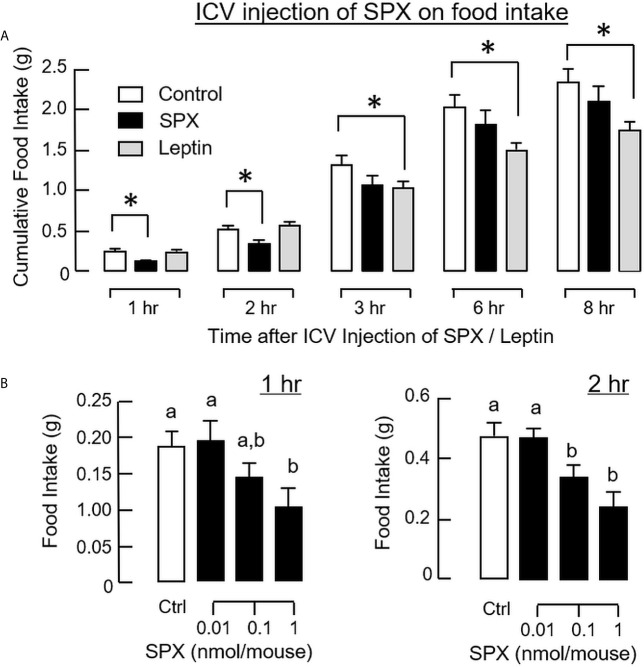
Central administration of SPX on food intake in mice. Time course **(A)** and dose dependence **(B)** for SPX treatment by ICV injection on food consumption. For time course study, SPX was tested at a dose of 1 nmol/mouse to examine its effects on food intake up to 8 h. For dose dependence, the corresponding effects of increasing levels of SPX (0.01-1 nmol/mouse) were monitored for 1 and 2 h, respectively. In these studies, ICV injection with artificial CSF was used as the control treatment and parallel injection with leptin (1 nmol/mouse) was conducted as a positive control for feeding response. The groups denoted by asterisks (*)/different letters represent a significant difference at *p* < 0.05.

**Figure 7:**
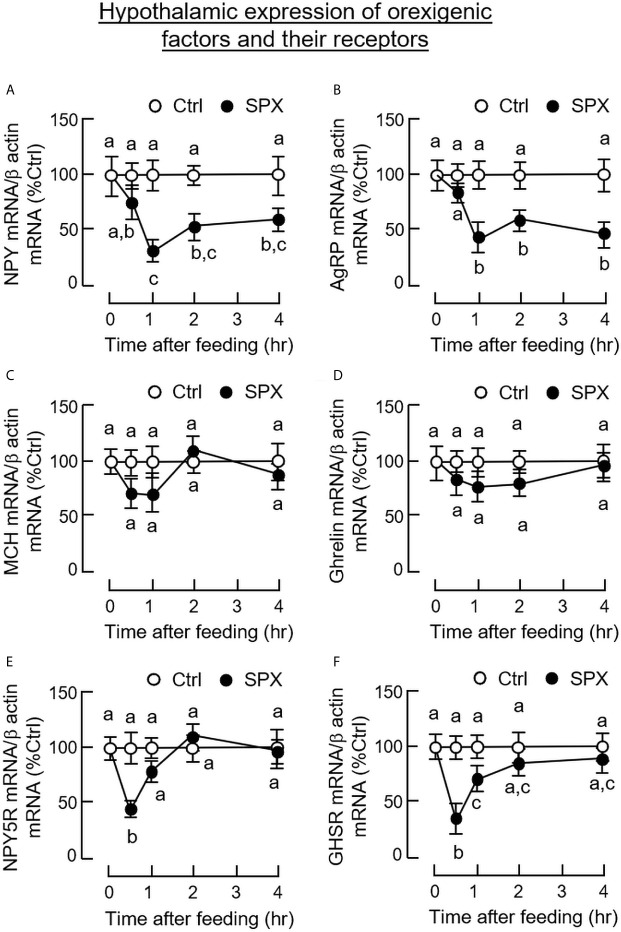
Central administration of SPX on hypothalamic expression of orexigenic factors and their receptors. ICV injection of SPX (1 nmole/mouse) was conducted in mice with parallel injection of artificial CSF as control treatment. The hypothalamus was harvested at the time points as indicated and used for total RNA isolation followed by real-time PCR for transcript expression of NPY **(A)**, AgRP **(B)**, MCH **(C)**, ghrelin **(D)**, NPY5R **(E)** and GHSR **(F)**, respectively. In this study, β actin mRNA was used as the internal control. The groups denoted by different letters represent a significant difference at *p* < 0.05.

**Figure 8:**
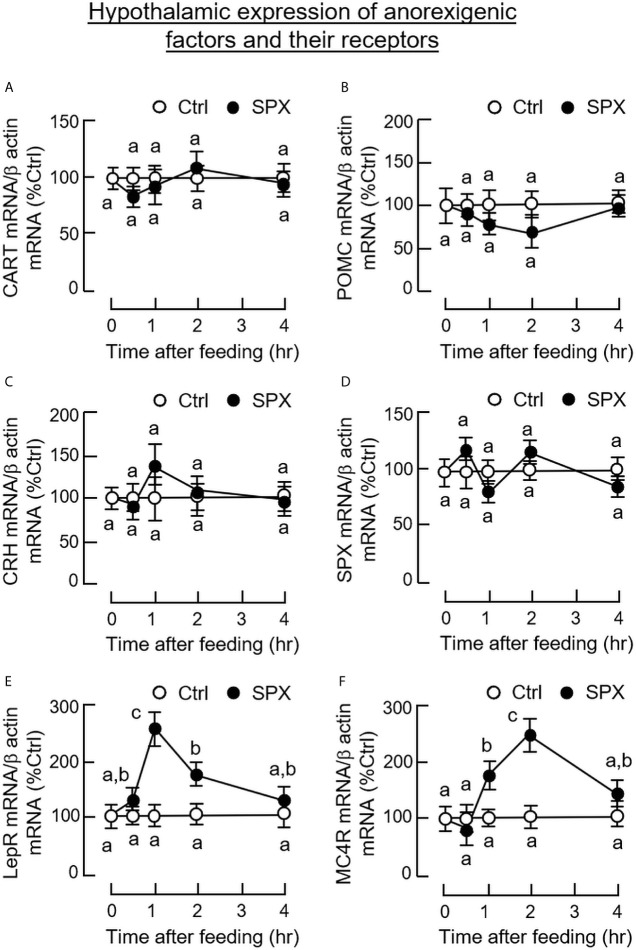
Central administration of SPX on hypothalamic expression of anorexigenic factors and their receptors. ICV injection of SPX (1 nmole/mouse) was conducted in mice with parallel injection of artificial CSF as control treatment. The hypothalamus was harvested at different time points and used for total RNA isolation followed by real-time PCR for transcript expression of CART **(A)**, POMC **(B)**, CRH **(C)**, SPX **(D)**, LepR **(E)** and MC4R **(F)**, respectively. In this study, β actin mRNA was used as the internal control. The groups denoted by different letters represent a significant difference at *p* < 0.05.

To confirm that the feeding regulation by SPX was mediated by central actions acting within the brain, the mice was subjected to ICV injection of SPX (1 nmol/mouse) at the beginning of the dark phase (at 10:00 am) similar to the preceding study. Again, a transient drop in food consumption was noted within the first 2 h after the initiation of feeding period (**Figure 6A**). Although a similar reduction in food intake was also observed in the group with ICV injection of leptin (the positive control for the feeding response), the inhibitory action could be noted after 3 h and maintained up to 8 h in the dark phase. Similar to IP injection, increasing doses of SPX could trigger a drop in food consumption in a concentration-related fashion during the first 2 h after ICV injection (**Figure 6B**). For the central actions of SPX on hypothalamic expression of orexigenic/anorexi-genic signals, ICV injection of SPX was effective in attenuating NPY (**Figure 7A**) and AgRP mRNA levels up to 4 h (**Figure 7B**) but did not modify the gene expression of other orexigenic factors including MCH (**Figure 7C**) and ghrelin (**Figure 7D**). Similarly, SPX treatment was found to have no effects on transcript levels of anorexigenic factors, including CART (**Figure 8A**), POMC (**Figure 8B**), CRH (**Figure 8C**), and SPX (**Figure 8D**). In the same study, however, differential regulation of the receptors for orexigenic and anorexigenic signals could still be noted in the hypothalamus. In this case, a transient drop in NPY5R (**Figure 7E**) and GHSR mRNA levels (**Figure 7F**) with peak responses occurred at 30 min was observed with a delayed elevation in transcript expression of leptin receptor (LepR, **Figure 8E**) and melanocortin 4 receptor (MC4R, **Figure 8F**) after 1 h with SPX treatment. Of note, leptin gene expression was not detectable in the samples prepared from the hypothalamus in our study.

## Discussion

SPX is known to be a pleiotropic peptide with diverse functions and widely expressed at tissue level (see introduction for details). In human studies, a drop in serum SPX/tissue SPX expression (e.g., in omental and subcutaneous fat) has been associated with obesity (14, 46), especially for adult (24) and childhood obesity (22, 23). In rodents with diet-induced obesity, SPX treatment was shown to reduce body weight (13, 14) presumably by decreasing caloric intake and FFA absorption with a parallel rise in locomotor activity during the dark phase (14). However, the functional role of SPX in appetite control and the mechanisms involved have not been explored in these previous studies. To shed light on the role of SPX as a satiety factor in mammalian model, feeding experiment was conducted in mice to test if food intake could induce SPX signal in circulation and/or at the tissue level. In our study, food consumption during the dark phase was shown to trigger a transient rise in serum SPX in mice with differential modulation of SPX expression in omental fat and glandular stomach. Similar to serum SPX, SPX mRNA expression in glandular stomach exhibited a rapid and transient elevation with parallel increase in tissue SPX content. As reviewed by IHS staining, the rise in protein signals for SPX in glandular stomach occurred mainly in the gastric mucosa, especially in the surface layer with foveolar cells and in the main body of the gastric glands composed of parietal cells and chief cells. In the case of omental fat, a transient rise in SPX transcript expression was noted during the dark phase without food provision and this effect could be reduced by food intake. Despite the responses in transcript expression, no notable changes in tissue content/immunostaining of SPX were observed in omental fat. Out of the 10 tissues selected for our study, the omental fat and glandular stomach were the only two with SPX responses after feeding while the other tissues examined did not show any significant changes in SPX gene expression (the same is also true for SPX tissue content/immunostaining in some tissues, e.g., the liver and forestomach). Apparently, unlike the widespread distribution of SPX signals revealed by our preceding study (see Part I of this three-paper series), the postprandial responses of SPX in the mouse are tissue-specific and highly restricted to a limited no of target tissues.

In our feeding experiment, our results demonstrated that SPX signals in mouse (both in circulation and in glandular stomach) were inducible by food intake. Compared to the studies in fish species (e.g., goldfish (9)), the postprandial response of SPX in circulation (i.e., the endocrine component of SPX signals) is well conserved while the corresponding responses occurring at the tissue level (36) appear to be quite different. In goldfish, the tissue responses of SPX induced by feeding can be divided into (i) a peripheral component in the liver, which acts as a major source of SPX released into circulation (36); and (ii) the central component in the brain with SPX up-regulation in brain areas involved in appetite control including the telencephalon, hypothalamus, and optic tectum (9). Unlike the fish models with feeding circuitry spreading among different brain areas (37), the hypothalamus, including the ARC and PVN, represents a key structure in mammals for central regulation of feeding behaviors and energy homeostasis (43). In our study with the mouse, the postprandial rise in SPX signals was not apparent in the liver and hypothalamus, despite the feeding responses for NPY in the hypothalamus (47), leptin in omental fat (48), and ghrelin in the stomach (49) could still be observed after food intake. Our results suggest that (i) the central response of SPX may be missing in the mouse/occurred in brain areas other than the hypothalamus, and (ii) the liver may not be a major organ in the mouse for the peripheral responses of SPX induced by feeding. In previous reports for SPX expression in cell lines (e.g., β-TC3 and COS-7 cells), SPX was located in secretory vesicles and confirmed to be released into culture medium (1, 21), presumably after protein processing in Golgi and endoplasmic reticulum (21). These findings imply that SPX expression can be correlated with SPX secretion at the cellular level. Given that (i) up-regulation of SPX mRNA level in glandular stomach was noted simultaneously if not preceding the corresponding response in serum SPX, (ii) the postprandial rise of SPX mRNA expression in glandular stomach also occurred with a parallel elevation in SPX protein content, and (iii) the protein signals of SPX induced by feeding were identified in the foveolar cells (for mucus release), parietal cells (for HCl secretion) and chief cells (for enzyme production) within the gastric mucosa of glandular stomach, it is tempting to speculate that (i) SPX may have local actions in the stomach for digestive functions (presumably by acting at the level of gastric glands), and (ii) the glandular stomach may be a key structure in the mouse with the peripheral responses of SPX and contribute to the rise in serum SPX induced by food intake. In our study, the omental fat was shown to have opposite changes in transcript expression of SPX and leptin in the unfed group, and interestingly, the transient rise in SPX signal in the same tissue caused by food deprivation could be suppressed by food intake. Although our results indicate that SPX expression in omental fat is sensitive to the feeding status, the functional relevance of these findings is still unclear. In human studies, a negative correlation of serum SPX with leptin signal in circulation is well-documented, e.g., in obese females (24) and children/adolescents diagnosed to be overweight (23, 28). In a recent report in obese girls with bariatric surgery, a gradual rise in serum SPX was detected with parallel drop in leptin secretion together with notable improvement in BMI and CVD risk factors (50). These findings, taken together, raise the possibility that interaction between SPX and leptin may play a role in regulating body weight/energy homeostasis.

For our studies in mouse model, the functional role of SPX in appetite control is particularly interesting as (i) SPX is known to be co-evolved with galanin in the same gene lineage (42), and (ii) galanin can serve as a feeding stimulator (e.g., in rodents) by up-regulating NPY signal with a parallel drop on leptin sensitivity at the hypothalamic level (51). In mammals, the biological actions of galanin are mediated by 3 subtypes of galanin receptors, namely GalR1-3, functionally coupled to the cAMP/PKA-, PLC/PKC- and Ca^2+^-dependent pathways (52) and food intake induced by galanin is mediated by GalR1 in the brain with differential modulation of neurotransmission *via* the 5HT_1A_ and α2 adrenergic receptors in the hypothalamus (53). Based on functional expression studies, GalR2 and GalR3 have been confirmed to be the cognate receptors for SPX (3, 42) and this idea is also supported by our NMR docking analysis for receptor binding of mouse SPX (part I of this three-paper series), in which SPX was shown to associate with GalR2 and GalR3 *via* specific interactions with the well-conserved residues in TMD_2&7_ and ECL_1&2_ domains of the two receptors. However, the functional role of GalR2 and GalR3 in feeding regulation by SPX is still unclear. In our study with the mouse, peripheral administration (by IP injection) or central administration of SPX (by ICV injection) were both effective in triggering a transient inhibition on food intake during the first couple of hours in the dark phase. This inhibitory effect also occurred with parallel attenuation of NPY and AgRP transcript expression in the hypothalamus but with no corresponding changes in gene expression for other orexigenic (e.g., MCH and ghrelin)/anorexigenic factors (e.g., POMC, CART, and CRH). In the same study, hypothalamic expression of LepR and MC4R were elevated with parallel drops in NPY5R and GHSR signals, suggesting that differential regulation of the receptors for orexigenic and anorexigenic factors could also be induced by SPX. Similar to the rat (54), GalR2 and GalR3 expression, both at the transcript and protein levels, could be detected in the hypothalamus of the mouse. In the mouse model, feeding inhibition by SPX was not mimicked by the GalR2 agonist dN1-Qu but could be abolished by co-treatment with the GalR3 antagonist SNAP. However, parallel treatment with the GalR2 antagonist M871 was not effective in this regard. In the studies with GalR2/3 antagonists, the same was also true for the corresponding inhibition by SPX on hypothalamic expression of NPY, AgRP, NPY5R, and GHSR. These findings, as a whole, suggest that feeding inhibition by SPX observed in the mouse was caused by central inhibition of orexigenic signals (NPY and AgRP) with parallel modulation of the sensitivity for feeding stimulators/inhibitors at the receptor level (i.e., NPY5R and GHSR down-regulation with elevated LepR and MC4R expression), and probably, the regulatory effects by SPX were mediated by GalR3 activation within the hypothalamus.

The feeding inhibition by SPX *via* GalR3 activation revealed by the present study implies that SPX and galanin may form an intricate system in the brain for appetite control. In previous studies, SPX was shown to activate GalR2 and GalR3 but not GalR1 expressed in HEK293 cells (42). Activation of GalR2a and GalR2b (the functional equivalence of GalR3 in fish models) with a similar lack of GalR1 cross-reactivity by SPX have been recently confirmed in GalR1 and GalR2 of fish origin (e.g., tilapia) (55), suggesting that the receptor specificity of SPX is well-conserved in vertebrates. In mammals, galanin is a 29-30 a.a. gut-brain peptide with a strong preference for GalR1 and GalR2 but not GalR3 for receptor binding/activation (56). Its stimulatory effect on food intake *via* GalR1 activation is well documented (51, 52) and probably mediated by inducing NPY release in the hypothalamus (57). Of note, galanin-like peptide (GALP), another member of the galanin family with a binding selectivity for GalR2 over GalR1 (52), has been reported to stimulate food intake *via* GalR2 activation and the effect was mediated by NPY neurons in the DMN of the hypothalamus (58). These findings, together with ours, indicate that the gene products of the SPX/galanin gene lineage, namely SPX, GALP and galanin, may form an intrinsic component for central regulation of feeding through differential activation of the three subtypes of galanin receptors expressed in the hypothalamus. Given that galanin regulation of NPY and orexin neurons can be modified by leptin (57, 59) and feeding inhibition by leptin reciprocally can be blunted by GalR2 activation in the hypothalamus using galanin analogs (60), the possibility of SPX interaction with leptin in appetite control cannot be excluded. Although leptin was shown to reduce food intake in our studies and SPX was effective in stimulating LepR expression in the hypothalamus, SPX inhibition of feeding caused by secondary induction of leptin signals is rather unlikely as the feeding inhibition by leptin had a slow onset (after 3 h) with prolonged action (up to 24 h), which is opposite to the rapid (≤ 1 h) but transient effect of SPX (for 2–3 h). Besides the feeding responses, SPX could also reduce body weight similar to previous reports in obese mice induced by high fat diet (13, 14). In our study, despite the inhibition observed during the first couple of hours in the dark phase, the 24-h cumulative food intake was not affected by SPX treatment, implying that compensatory feeding might have occurred during the latter half of dark phase and/or during the light phase. Apparently, the inhibitory effect on weight gain by SPX was not mediated by a drop in caloric intake, and probably, it might be the result of reducing FFA uptake at tissue level (13) with parallel elevation of locomotor activity during the dark phase as reported previously (14).

**Figure 9:**
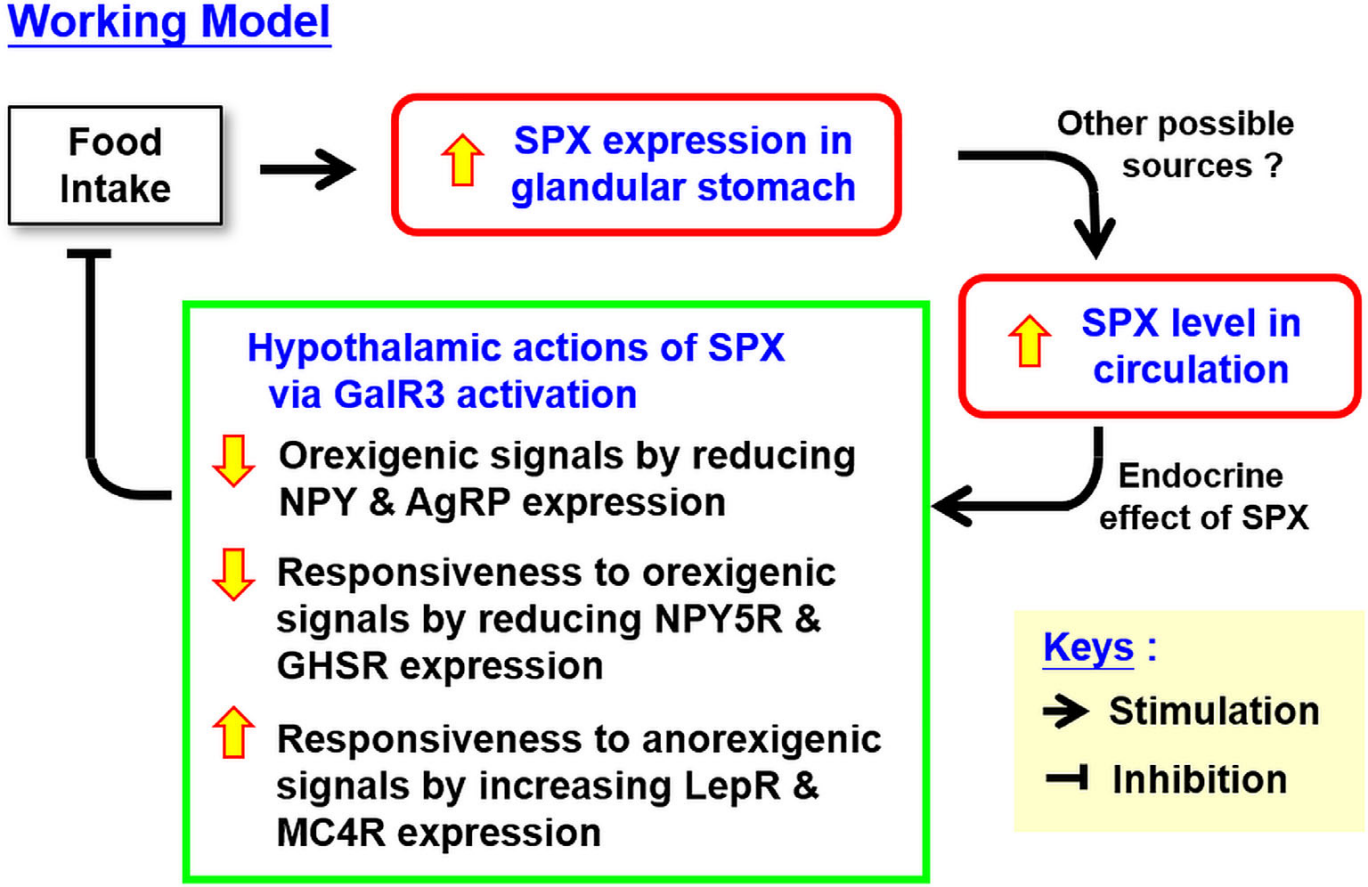
Working model for the role of SPX as a satiety factor in the mouse *via* regulatory actions within the hypothalamus. In the mouse, food intake can induce SPX gene expression in glandular stomach and the subsequent rise in SPX production may contribute to the elevation of SPX signal in blood observed after feeding. The circulating SPX by acting in an endocrine manner can initiate a feedback inhibition on food intake by central actions acting in the hypothalamus. In this case, hypothalamic expression of orexigenic factors (including NPY and AgRP) and receptors for orexigenic signals (including NPY5R and GHSR) can be suppressed by SPX with concurrent up-regulation of the receptors for anorexigenic signals (including LepR and MC4R). The drops in orexigenic signals together with the differential regulation of hypothalamic responsiveness to orexigenic and anorexigenic factors probably can lead to feeding inhibition in the mouse. In the model proposed, we do not exclude the possibility that the postprandial rise of SPX in circulation may also be originated from other tissues in addition to the glandular stomach.

In summary, using the mouse as an animal model, food intake was shown to up-regulate SPX signal in circulation with parallel rises of SPX transcript and protein levels in the glandular stomach. Meanwhile, a drop in SPX transcript level without a corresponding change in protein signal was observed in omental fat. In parallel studies, SPX treatment by peripheral (IP) or central (ICV) administration were both effective in reducing food intake during the initial period of the dark phase. This inhibitory effect occurred with rapid reduction of NPY, NPY5R, AgRP. and GHSR expression with concurrent up-regulation of LepR and MC4R in the hypothalamus. Using a pharmacological approach, SPX inhibition on food intake and hypothalamic expression of NPY, NPY5R, AgRP, and GHSR were also confirmed to be mediated by GalR3 but not GalR2 activation. Based on our findings, a working model has been proposed for the functional role of SPX as a satiety factor *via* its regulatory actions acting at the hypothalamic level (**Figure 9**). In this model, food intake can trigger a rapid activation of SPX expression in the glandular stomach and the subsequent rise in SPX protein level also contributes to the postprandial elevation of SPX signal in circulation. Through endocrine action, SPX in circulation can activate GalR3 in the hypothalamus. As a result, hypothalamic expression of orexigenic signals including NPY and AgRP was suppressed. Meanwhile, differential regulation of the responsiveness for both orexigenic and anorexigenic signals *via* modulation of the respective receptors also occurred at the hypothalamic level. In this case, the receptors for orexigenic factors including NPY5R (for NPY) and GHSR (for ghrelin) were down-regulated with parallel elevations in receptor expression for anorexigenic factors including LepR (for leptin) and MC4R (for melanocortin). The hypothalamic actions of SPX through GalR3 activation, as a whole, can lead to inhibition of food intake, which may form the basis of a feedback loop involved in the satiation response in the mouse after a meal. In the model proposed, the contribution of circulating SPX from tissues other than the glandular stomach cannot be excluded and the functional link between food intake and SPX expression in glandular stomach is still unclear. Given that (i) insulin release induced by glucose uptake in goldfish was shown to be a potent stimulant for SPX expression in the liver, which can contribute to the postprandial rise of SPX in circulation in the same species (36), and (ii) modification of SPX expression and secretion by insulin treatment has also been reported in *in vitro* culture of porcine islets (61), the postprandial signal of insulin in the SPX responses observed in the mouse model for sure can be a logical follow-up for our study on feeding control by SPX. The drop in SPX signal detected in omental fat after feeding as well as the opposite changes in transcript expression of SPX and leptin also raise the possibility that SPX may play a role in hormone regulation/production in white adipose tissue, which is still an unexplored area waiting to be investigated.

## Data Availability Statement

The original contributions presented in the study are included in the article/**Supplementary Material**. Further inquiries can be directed to the corresponding author.

## Ethics Statement

The animal study was reviewed and approved by The Committee on the Use of Live Animal in Teaching and Research, The University of Hong Kong, Hong Kong.

## Author Contributions

AW was the PI and grant holder. AW, MW, and MH were responsible for project planning and data analysis. MW and YC were in charge of the feeding experiment and studies with IP and ICV injection. MH and CL were responsible for the GalR2/3 agonist/antagonist study to define the receptor specificity of SPX actions. MW and MH were both involved in IHS staining for SPX expression at the tissue level. Manuscript preparation was done by AW and ZB. All authors contributed to the article and approved the submitted version.

## Funding

This study was supported by HMRF grant (13142591), Food and Health Bureau (HKSAR), and GRF grants (17105819, 17113918, and 17117716), Research Grant Council (HK).

## Conflict of Interest

The authors declare that the research was conducted in the absence of any commercial or financial relationships that could be construed as a potential conflict of interest.

## Abbreviations

SPX, Spexin; GALP, Galanin-like peptide; NPY, Neuropeptide Y; AgRP, Agouti-related neuropeptide; GHSR, Ghrelin receptor; NPY5R, NPY type 5 receptor; LepR, leptin receptor; MC4R, melanocortin 4 receptor; IP injection, intraperitoneal injection; ICV injection, intracerebroventricular injection; IHS staining, immuno-histochemical staining; CSF, Cerebrospinal fluid; ORF, Open reading frame; FFA, free fatty acid; ARC, arcuate nuclei; PVN, paraventricular nuclei; DMN, dorso-medial nuclei; CVD, Cardiovascular disease; BMI, body mass index; TMD, trans-membrane domain; ECL, Extracellular loop; CNS, Central nervous system.
