# Supplementary material for: Mouse Spexin: (II) Functional Role as a Satiety Factor inhibiting Food Intake by Regulatory Actions Within the Hypothalamus
Source: Front Endocrinol (Lausanne). 2021 Jul 2;12:681647. doi: 10.3389/fendo.2021.681647 (PMC8283969; doi:10.3389/fendo.2021.681647)
Supplement: Supplementary file 1 [file DataSheet_1.pdf]

Supplemental Fig.1

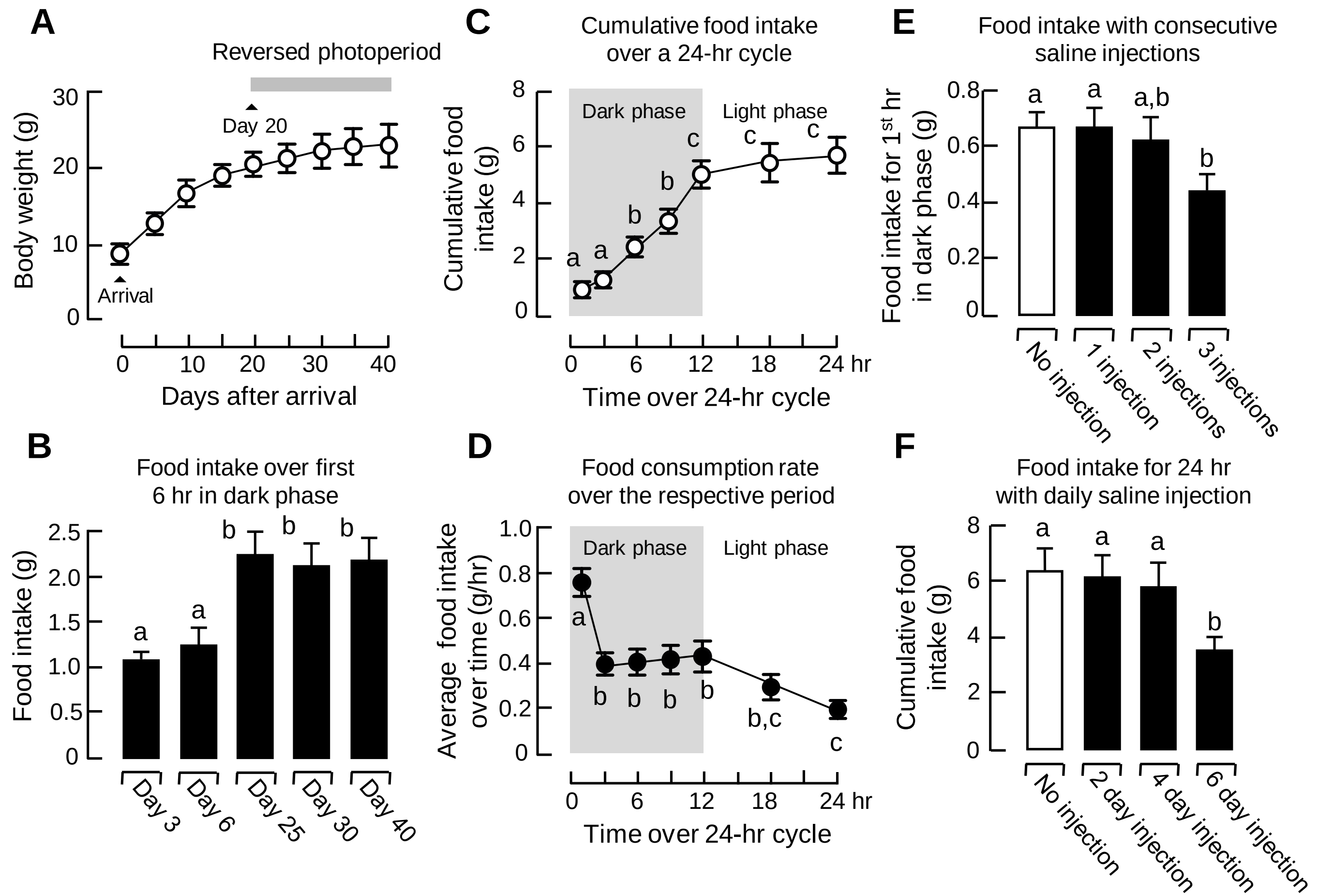

Validation of feeding performance and body growth in mice acclimated to an inverted photoperiod. Change in body weight (A) and food intake during the first 6 hr in the dark phase (B) in mice during the acclimation to inverted photoperiod (12 hr dark:12 hr light, with light off and food provision at 10:00 AM). Cumulative food intake over a 24 hr cycle (C) and food consumption rate over the respective period (D) in mice acclimated to an inverted photoperiod (for ≥ 10 days). Effects of consecutive injections with saline in a single day (with a 5-min interval between injections starting at 10:00 AM) (E) or with daily injection of saline (with one injection/day at 10:00 AM) up to 6 days (F) on food intake in the mice acclimated to inverted photoperiod. Data presented are expressed as mean ± SEM (N = 6). For the data on food intake, statistical analysis was conducted using one-way ANOVA followed by Newman-Keuls test and the groups denoted by different letters represent a significant difference at $p < 0.05$.
