# Supplementary material for: Mouse Spexin: (II) Functional Role as a Satiety Factor inhibiting Food Intake by Regulatory Actions Within the Hypothalamus
Source: Front Endocrinol (Lausanne). 2021 Jul 2;12:681647. doi: 10.3389/fendo.2021.681647 (PMC8283969; doi:10.3389/fendo.2021.681647)
Supplement: Supplementary file 2 [file DataSheet_2.pdf]

Supplemental Fig.2

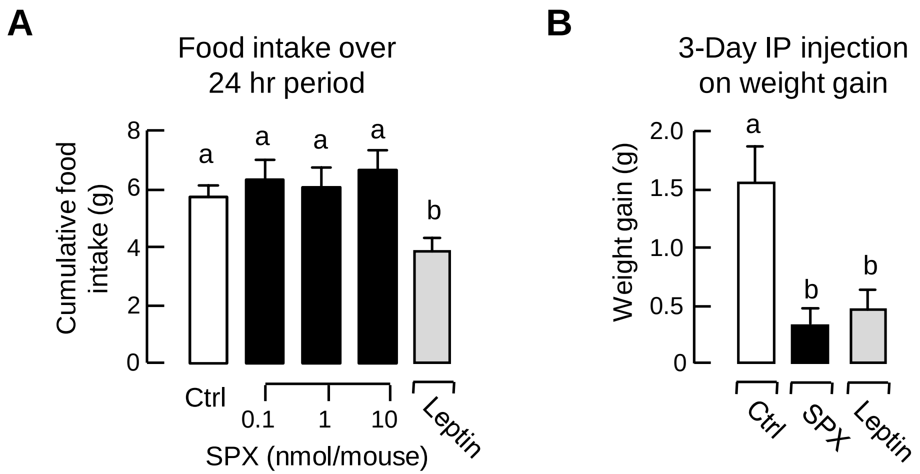

Effects of SPX on food intake and weight gain in mice acclimated to an inverted photoperiod. After acclimation to inverted photoperiod, the mice were subjected to IP injection (5 μl/g BW, administered at 10:00 AM) with increasing doses of SPX (0.1 - 10 nmol/mouse) to study the effect on cumulative food intake over a 24 hr period (A). In this experiment, leptin injection (10 nmol/ mouse) was also performed to serve as a positive control. In parallel study, mice were subjected to daily injection (IP) with SPX (10 nmol/mouse) or leptin (10 nmol/mouse) up to 3 consecutive days (one injection/day) to examine the effect on weight gain during the same period (B). In our experiments for the effects of SPX on food intake and weight gain, parallel injection with saline was used as the control treatment. Data presented (mean ± SEM, N = 8) denoted by different letters represent a significant difference at $p < 0.05$.
